# Precision Medicine in Type 1 Diabetes

**DOI:** 10.1007/s41745-023-00356-x

**Published:** 2023-03-07

**Authors:** Dominika A. Michalek, Suna Onengut-Gumuscu, David R. Repaske, Stephen S. Rich

**Affiliations:** 1 Center for Public Health Genomics, University of Virginia, Charlottesville, VA USA; 2 Department of Public Health Sciences, University of Virginia, Charlottesville, VA USA; 3 Division of Endocrinology, Department of Pediatrics, University of Virginia, Charlottesville, VA USA

**Keywords:** Precision medicine, Type 1 diabetes, Precision diagnostics, Precision therapeutics, Precision prognostics, Precision prevention, Precision monitoring

## Abstract

Type 1 diabetes is a complex, chronic disease in which the insulin-producing beta cells in the pancreas are sufficiently altered or impaired to result in requirement of exogenous insulin for survival. The development of type 1 diabetes is thought to be an autoimmune process, in which an environmental (unknown) trigger initiates a T cell-mediated immune response in genetically susceptible individuals. The presence of islet autoantibodies in the blood are signs of type 1 diabetes development, and risk of progressing to clinical type 1 diabetes is correlated with the presence of multiple islet autoantibodies. Currently, a “staging” model of type 1 diabetes proposes discrete components consisting of normal blood glucose but at least two islet autoantibodies (Stage 1), abnormal blood glucose with at least two islet autoantibodies (Stage 2), and clinical diagnosis (Stage 3). While these stages may, in fact, not be discrete and vary by individual, the format suggests important applications of precision medicine to diagnosis, prevention, prognosis, treatment and monitoring. In this paper, applications of precision medicine in type 1 diabetes are discussed, with both opportunities and barriers to global implementation highlighted. Several groups have implemented components of precision medicine, yet the integration of the necessary steps to achieve both short- and long-term solutions will need to involve researchers, patients, families, and healthcare providers to fully impact and reduce the burden of type 1 diabetes.

## Introduction

A typical definition of ‘precision medicine’ includes the concept of targeting disease prevention and treatment based upon individual characteristics, including genetics, environments (exposures) and lifestyles–the “right treatment to the right person at the right time”. The precision medicine approach is contrasted to the “one size fits all” or “average patient” concept that, in truth, may not be the typical practice of modern medicine. Nonetheless, precision medicine forms a framework for integrating many sources of information to better guide approaches for the improvement of individual and public health.^1^

The implementation of precision medicine in type 1 diabetes will be a function of several key components. First, there are advances in medical science that include genomics,

**Genomics**: is the study of all of a person’s genes (the human genome), in contrast to genetics, which focuses on one (or a subset) of genes. Genomics also includes the interactions of genes and the individual’s environment.

imaging, miniaturization, drug delivery, and development of biomarkers and therapeutics. Second, the field of data science has incorporated mining of health care data from electronic health record systems with advanced analytics, including artificial intelligence, machine and deep learning approaches, and neural networks. Third, there is recognition of the impact of lifestyle and social inequities as a major driver of health and disease. Finally, national policies on health care and vast variation in global wealth and public health approaches on precision medicine has significant impact on how readily and effectively precision medicine can be implemented. Given the appropriate framework of implementation of precision medicine, the underlying heterogeneity of type 1 diabetes

**Type 1 diabetes**: is a chronic disease in which the insulin-producing beta cells in the pancreas are destroyed by the individual’s immune system, requiring exogenous insulin for survival. Type 1 diabetes was once known as “juvenile diabetes” or “insulin-dependent diabetes” as it was thought to be a disease of children; however, nearly one-half of those with type 1 diabetes develop the disease in adulthood.

and overlap with other forms of disease will need to be addressed before true benefits of science and medical care can be applied globally.

## Type 1 Diabetes

Type 1 diabetes is considered to be an autoimmune disease in which the etiology is due to destruction of insulin-producing pancreatic beta cells by the host immune system in response to a foreign antigen triggering a response in a genetically susceptible individual.^2^ Although type 1 diabetes is often viewed as a disease of children and young adults, it can occur at any age and in people of diverse genetic ancestry. Factors that influence the risk of type 1 diabetes include genetic predisposition, with genetic factors accounting for ~ 50% of the overall risk.^3^ While recent research has discovered the majority of genetic factors contributing to risk and many of their putative functional targets,^4,5^ these findings are based predominantly on studies in European ancestry with disease occurring during childhood. Nearly one-half of the genetic risk is assigned to genes in the human Major Histocompatibility Complex (MHC), primarily the HLA

**HLA genes**: are located in the human genome within the Major Histocompatibility Complex (MHC) on the short arm of chromosome 6 (6p21.3), along with over 100 other genes. HLA (Human Leukocyte Antigen) genes code for proteins that play an essential role in immunity and are composed of HLA class I (HLA-A, HLA-B, HLA-C) and HLA class II (HLA-DR, HLA-DQ, HLA-DP) with extensive genetic variation.

class II and class I genes^6,7^; however, within these key risk genes there is allelic variation associated with risk across populations, even when restricted to European ancestry groups.^8^ There is evidence that there are ancestry-specific loci and variants contributing to type 1 diabetes genetic risk, making risk prediction dependent upon site and potentially limiting transferability of genetic risk score derived from one population to another.^8,9^

The development of type 1 diabetes in most individuals can be missed, despite symptoms that include increased thirst and urination, increased hunger, blurred vision, fatigue, and unexpected weight loss. Many of these symptoms are initially mild, and can masquerade as common diseases of childhood.^2^ In children, the signs of type 1 diabetes result from the increase in glucose in the blood, triggering both osmotic removal of fluid from tissue and osmotic diuresis (which in turn increases thirst) and loss of insulin that is critical for entry of glucose into the cells of the body where it can be metabolized into energy (fatigue). As insulin deficiency progresses, the liver produces alternate fuels, the ketones, that do not require insulin for entry into cells but are acidic leading to potentially deadly diabetic ketoacidosis (DKA)

**Diabetic ketoacidosis**: (DKA) is a serious complication of diabetes that develops when there is insufficient insulin produced (through destruction of beta cells) and fat is used for fuel, with a subsequent accumulation of ketones, causing the blood to become acidic and leading to toxicity in all tissues (including brain, heart, muscle) of the body..

Typically, ~ 5% of those who develop type 1 diabetes have a parental history of the disease, although the rates vary by population, with the 9% occurring in the high prevalence Finnish population.^10^ Across multiple populations of European ancestry, the fathers with type 1 diabetes more frequently transmit disease to offspring than mothers^10,11^; however, there is no family history and often no family awareness of the early symptoms of type 1 diabetes in 90–95% of childhood cases. Children are first recognized with the development of DKA in ~ 40% of new onset cases, but precision prediction of type 1 diabetes followed by intensive monitoring for symptoms represents a critical area for mitigation of life-threatening illness. Reduction of DKA to nearly 0% is possible with early recognition and monitoring and with potential to preserve beta cell function and delay disease onset.^12–14^

Implementation of precision medicine in type 1 diabetes is tied necessarily to the natural history of the disease. Type 1 diabetes is thought to consist of an initiation of the immune system attacking, modifying, impairing, and (ultimately) destroying, the insulin-producing beta cells,^15–17^ with the “triggering event” unknown at this time but could include a variety of environmental factors. It should be noted, however, that the progression of the attack (as measured by presence of islet autoantibodies) differs across individuals, in terms of the rate of beta cell loss in the “pre-diabetic” period.^18^ Thus, the decline in beta cell mass can be represented as “waves” of effector and regulatory T cells mediating beta cell destruction, increasing in intensity over time, with the overall progression in any individual determined by numerous factors and their effect on the immune system.^18^ As shown in the TEDDY study, the progression from a single islet autoantibody to multiple autoantibodies (and clinical disease) is associated with a number of factors, including which islet autoantibody appears first.^19^

Since interventions in type 1 diabetes are dependent upon knowledge of initiation and progression to clinical disease, a major barrier for immune intervention in the application of precision medicine to type 1 diabetes is the accurate prediction of risk. From a population perspective, the prediction of risk involves both genetic and islet autoantibody screening, with the fundamental question of whom to screen (and when to screen and whom to follow for islet autoantibodies).

## Principles of Precision Medicine

**Figure 1:**
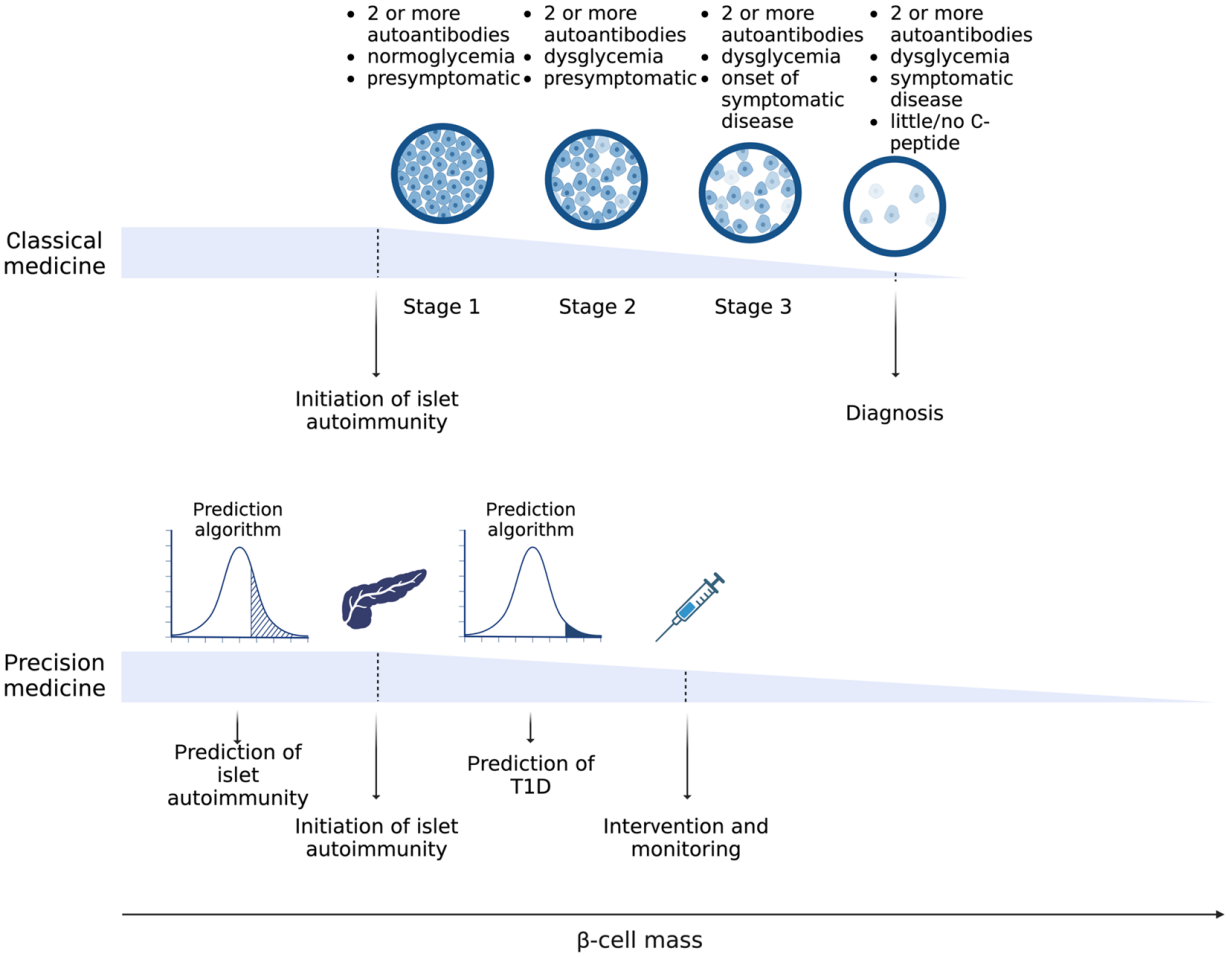
Contrast between “Classical medicine” and “Precision medicine” in type 1 diabetes (T1D), through prediction, therapeutics, and prevention.

The joint ADA/EASD Precision Medicine in Diabetes Initiative (PMDI) considered application and gaps in knowledge related to precision medicine “pillars” across multiple forms of diabetes,^20^ precision diagnosis, precision therapeutics, precision prevention, precision prognostics and precision monitoring (Fig. 1). Each of these areas was defined with identification of barriers to implementation and research gaps, with clarity on the differences in barriers and gaps relevant to type 1 diabetes as well as type 2 diabetes, monogenic

**Monogenic diabetes**: is a class of diabetes that is due to mutations in a single gene, in contrast to type 1 diabetes or type 2 diabetes that is partially due to many genetic factors and (unknown) environmental effects. Monogenic diabetes are rare, and includes neonatal diabetes mellitus (NDM) and maturity-onset diabetes of the young (MODY).

forms of diabetes and gestational diabetes

**Gestational diabetes**: (GDM) is a form of diabetes that can develop in women during pregnancy. Each year in the USA, GDM occurs in 2%-10% of women previously not diagnosed with diabetes, and results in an increased risk of subsequent development of type 2 diabetes..

These “pillars” differ in complexity across the forms of diabetes; for example, the genetic architecture of monogenic forms of diabetes is more advanced than for that of type 1 diabetes, and less advanced for type 2 diabetes. Within each form of diabetes, there remains heterogeneity in etiology, natural history and acknowledged differences that reflects incomplete knowledge of disease. Recent publications have further addressed precision medicine in specific applications^21–25^ as well as gaps in knowledge. It should be noted that many of the gaps in knowledge at the biostatistical and epidemiology perspective,^26^ such as spectrum bias, absence of appropriate biomarkers of risk, different levels of complexity, and individual variation in prognosis, treatment response and diagnostic accuracy, are applicable to all medical conditions.

### Precision Diagnosis

Precision diagnosis also incorporates the individual characteristics with predictors of disease (classical laboratory tests, novel biomarkers, genetic risk) that provides a temporal likelihood of risk, recognizing that a disease “evolves” over time and may be altered by other co-existing conditions/factors that also change over time. From a clinical perspective, precision diagnosis also could generate disease “subtypes”,

**Subtype**: or endotype, represents a distinct functional or physiological component of a disease or condition. Subtypes are used to identify more homogeneous groups of individuals that may have greater prediction of disease outcome or optimal use of a treatment.

especially in those situations that a subtype of a disease may have a preferred therapy or prognosis. In type 1 diabetes, the population characteristics include factors such as ancestry, age, the presence of islet autoantibodies,

**Islet autoantibodies**: are biomarkers of the autoimmune response in blood of those at risk of developing type 1 diabetes. The islet autoantibodies currently used for detection of risk bind to islet cells (ICA), glutamic acid decarboxylase (GADA), protein tyrosine phosphatase islet antigen-2 (IA2A), zinc transporter 8 (ZnT8A), and insulin (IAA).

and genetic risk (particularly HLA genotype) coupled with absence of C-peptide provides important clinical information for diagnosis.^19,27^ However, these predictors may not apply to all cases as (Fig. 1): there are cases diagnosed in adulthood (with a more indolent clinical course); the genetic risk score has low predictive value (given the low disease prevalence); there are differences in outcome associated with which islet autoantibody first emerges and the age at which it occurs; and there are those who have clinical feature of type 1 diabetes without islet autoantibodies or with “protective” genetic risk scores.^28^ Although type 1 diabetes is often considered as a homogeneous disease (complete destruction of beta cells leading to life-long insulin therapy), it is likely heterogeneous in etiology, clinical course, and factors that require subtyping for optimal (future) intervention and therapies.

### Precision Prevention

Precision prevention utilizes individual characteristics, coupled with their environment and individual preferences, to optimize outcomes of interventions for disease or disease risk. Interventions in diabetes can be varied, often pharmacological and behavioral (for type 2 diabetes) that can focus on reduced exposure to risk factors. Prevention is dependent, in part, on the presence of an intervention that is available, one that is economically accessible, with minimal off-target effects and globally available to ensure health equity.

In type 1 diabetes, the majority of prevention efforts involve development of immune interventions, including those involving beta cell preservation with T cell targets (teplizumab/anti-CD3,

**Teplizumab**: is a humanized anti-CD3 monoclonal antibody (under the brand name Tzeild) is the first and only approved treatment indicated to delay the onset of stage 3 type 1 diabetes in those with stage 2 (two or more islet autoantibodies and abnormal glucose tolerance) disease. Tzeild was approved by the U.S. Food and Drug Administration (FDA) on November 17, 2022.

and abatacept/anti-CD80 and anti-CD86),^29^ B cell targets (rituximab/anti-CD20), and inflammatory cytokines

**Inflammatory cytokines**: are a class of signaling molecules secreted from (predominantly) T helper cells (*T*_h_) and macrophages that are involved in the upregulation of inflammation.

(anti-TNF-alpha, anti-IL-6R).^29,30^ In addition, oral insulin or peptides.^31,32^ as well as a number of other targets as alternatives to islet transplantation have been investigated. A promising future intervention involves use of stem cells to augment residual beta cell reserve.^33^ Other approaches that include targeting processes to reduce inflammation involved in beta cell destruction through cytokine and free fatty acid sensitivity have been proposed, with protection of those cells from immune-mediated rejection.^34^

### Precision Therapeutics (or Precision Treatment)

Precision therapeutics utilizes the individual characteristics, including that of the disease state, to “tailor” treatment while minimizing adverse responses to the treatment.^20^ While there have been efforts to use genomic data (pharmacogenomics) to improve and target therapies, the primary approach to precision therapeutics is based on specific treatment guidelines based upon a diagnosis. Although much has been accomplished in pharmacogenetics and pharmacodynamics in diabetes, the focus has been primarily on type 2 diabetes and monogenic forms of diabetes, in which specific genotypes account for variability in response to a variety of treatment options.^24^

In type 1 diabetes, the only treatment option is insulin, yet there can be variation in the amount of insulin delivery, formulation of insulin, and monitoring of insulin and its physiologic effects through technological innovation; however, a pharmacogenomic approach to assess genotype-informed decision making on treatment has yet to be adopted, despite evidence that DNA sequence variants influence gene expression response to insulin via kinase modulators.^35^ While much is known about the genetic risk of developing type 1 diabetes, little is known about the genetic variation that modifies the response to insulin treatment; in addition, the genetic architecture that defines development of type 1 diabetes may not overlap with the ones that control insulin therapy. Greater understanding of the effect of insulin on signaling pathways and genetic modulation of effects may be important in tailoring optimal insulin administration guidelines.

### Precision Prognosis

Precision prognosis relates to risk of diabetes-related outcomes (complications)

**Diabetic complications**: represents the collection of target tissue damage organs due to the dysregulation of glucose. The major targets include the eye (retinopathy), nerve (neuropathy), kidney (nephropathy), foot (wound healing), heart (heart attack), and brain (stroke).

given an individual’s form (or subtype) of diabetes with their combination of biological (genetic), lifestyle, societal and cultural features. Implementation of precision prognosis includes prevalence of the type of diabetes in the population, availability and cost of diagnostic testing, understanding personal preferences for management and compliance, and risk of complications through predication and monitoring of systems. Prognosis, or prediction, of type 1 diabetes outcomes include optimizing quality of life and minimizing risk of complications (retinopathy, nephropathy, neuropathy, cardiovascular and cerebrovascular diseases).

In type 1 diabetes, there is increasing use of genetics (that accounts for ~ 50% of the risk) with genetic risk scores to distinguish type 1 diabetes from type 2 diabetes.^36^ as well as improved prediction in European ancestry populations.^37,38^ However, the genetic basis of complications of type 1 diabetes appears to be distinct from those variants associated with risk of type 1 diabetes itself, and the extent of heritability of the complications may vary. In most cases, presence of diabetes, of any form, is viewed as an independent risk factor for retinopathy, nephropathy, neuropathy, cardiovascular disease (myocardial infarction) and cerebrovascular disease (stroke). Further, glycemic control plays a major role in defining risk of complications from diabetes, particularly for retinopathy whether in type 1 diabetes.^39^ or type 2 diabetes,^40^ with little role for modification by genetic factors; however, gene set enrichment analyses of genome-wide association studies

**Genome-wide association studies**: (GWAS) utilize an array of genetic variants (typically 400,000 to 1,000,000 sites) that capture much of the common variation in the human genome that can be applied to cases (e.g., with type 1 diabetes) and controls (those without disease from the same ancestral population) to discover significant statistical associations.

in type 2 diabetes identified important biological pathways (lipid catabolism, digestion, mobilization and transport; nitric oxide biosynthesis; apoptosis; retinal ganglion cell degeneration) as targets for research.^41^ Recent results suggest that both glycemic and non-glycemic factors (HbA1c and BMI, psychological stress and cardiac autonomic neuropathy) are important in risk of neuropathy in type 1 diabetes.^42^ In contrast, the risk for nephropathy in type 1 diabetes^43^ or in combined type 1 and type 2 diabetes^44^ has been shown to have a strong genetic component. Further, a polygenic risk score for eGFR has been shown to be associated with incident kidney diseases and proteins related to kidney function.^45^

Cardiovascular and cerebrovascular diseases present the major causes of morbidity and mortality in type 1 diabetes.^46,47^ Although intensive glycemic control has been implicated as a primary risk reduction factor in type 1 diabetes,^48^ other factors also contribute to risk, including family history of cardiovascular and cerebrovascular disease, dyslipidemia, high blood pressure, smoking, and obesity.^49^ Even in absence of the traditional risk factors, cardiovascular disease, and particularly heart failure, is increased in type 1 diabetes, with rates greater than those observed in type 2 diabetes.^50^ Thus, accurately identifying those who are likely to progress to either single or multiple complications of type 1 diabetes could optimize management and decision making to treat risk factors of complications, rather than the specific complication. Other complications of type 1 diabetes include hypoglycemia

**Hypoglycemia**: is a condition in which the blood glucose is lower than that in the “normal range”. Hypoglycemia is often seen in type 1 diabetes from taking too much insulin, or not consuming sufficient quantities of carbohydrates for the insulin being taken, or timing of insulin administration and physical activity. Severe hypoglycemia may lead to loss of consciousness and seizures.

and fatty liver disease, each with specific risk factor interventions and monitoring efforts to affect outcome.

### Precision Monitoring

Precision monitoring is based upon the collection of biological, behavioral and environmental data that can reflect the temporal status of the individual, their disease state and their response to intervention/treatment. Early monitoring activities were restricted to measuring blood glucose levels and HbA1c

**Hemoglobin A1c**: (HbA1c) is a blood test that measures chronic, or average, level of blood sugar over the past few months. For those without diabetes, the normal HbA1c is typically 4%-5.6%, with levels 5.7–6.4% considered “prediabetes”, and HbA1c greater than 6.5% is seen more in those with diabetes. Consistent high values of HbA1c are considered as risk for complications of diabetes.

as surrogates for the individual’s changing physiology and response to insulin injections. With advancement in technology, these characteristics of glucose homeostasis and other physiologic characteristics can be obtained through digital applications, sensors, assays and novel technologies. Digital technologies have the potential to provide accurate and clinically relevant information to improve glycemic control, with significant efficacy shown by use of continuous glucose monitoring in both type 1 diabetes and type 2 diabetes.^51^

## The Increasing Global Burden of Type 1 Diabetes

Although type 1 diabetes accounts for a relatively small proportion of all forms of diabetes (including the majority, type 2 diabetes, but also monogenic/neonatal forms, gestational, and atypical diabetes), the incidence of type 1 diabetes appears to be increasing globally.^52^ The increase in type 1 diabetes is not likely due to genetic factors, as the rates of change of frequency of risk alleles that account for the observed increase, even under the most favorable modes of inheritance, would take hundreds of generations, not the few generations observed. The SEARCH for Diabetes in Youth study reported that the adjusted annual incidence of type 1 diabetes across five centers in the USA increased by 1.8% per year in those 0–19 years of age.^53^ The increase in incidence, however, was not uniform across genetic ancestries, with the adjusted annual increase in non-Hispanic whites (1.4%) less than non-Hispanic black (2.2%), Asian/Pacific Islander (3.7%) or Hispanic (4.2%) participants. The increase in annual incidence in non-Hispanic whites (1.4%) was less than the 3.4% annual rate of increase in type 1 diabetes reported by the larger EURODIAB study (84,000 children aged 0–14 years from 22 countries) over a longer observation period (1989–2013).^54^

In an updated, global analysis and modeling of prevalence

**Prevalence**: is the proportion of individuals in a defined population who are diagnosed with a disease (or attribute) at a specific point in time. Prevalence of type 1 diabetes differs by population and has exhibited different rates of increase over time. In type 1 diabetes, the prevalence in European-ancestry populations has been estimated at ~4/1000, although variation by country exists, with lower (but increasing prevalence) in non-European-ancestry populations.

and incidence

**Incidence**: is the rate of new cases of a disease (or attribute) occurring in a specific population over a defined period of time (often a year). In type 1 diabetes, the incidence in European-ancestry populations has been estimated at ~13/100,000 per year, with variation by country and ancestry.

rates estimated from 94 countries as mined from the literature, it was shown that high-income countries (with 17% of the global population) accounted for nearly one-half of the incident cases.^55^ Asia (with 60% of the global population) had the largest number of incident cases (32% of the total) while Europe (with 10% of the global population) had 27% of the total incident cases.^55^ The distribution of incident cases varied by country, as well as by age. Together, these and other studies.^56^ suggest that genetic factors, while important in defining risk of type 1 diabetes, is not driving the observed increase in incidence (and prevalence) globally. More likely, the increase in type 1 diabetes incidence could be accounted by improvement in clinical diagnosis/detection, and the interaction of genetic risk with changing environmental exposure that manifest in epigenetic modification. Thus, application of precision medicine approaches to type 1 diabetes should account for these factors, and address the gaps in knowledge across genetic ancestry, access to health care, and differences in non-genetic factors that influence risk of developing type 1 diabetes and risk of complications.^20^

## Implementing Precision Medicine in Type 1 Diabetes

**Figure 2:**
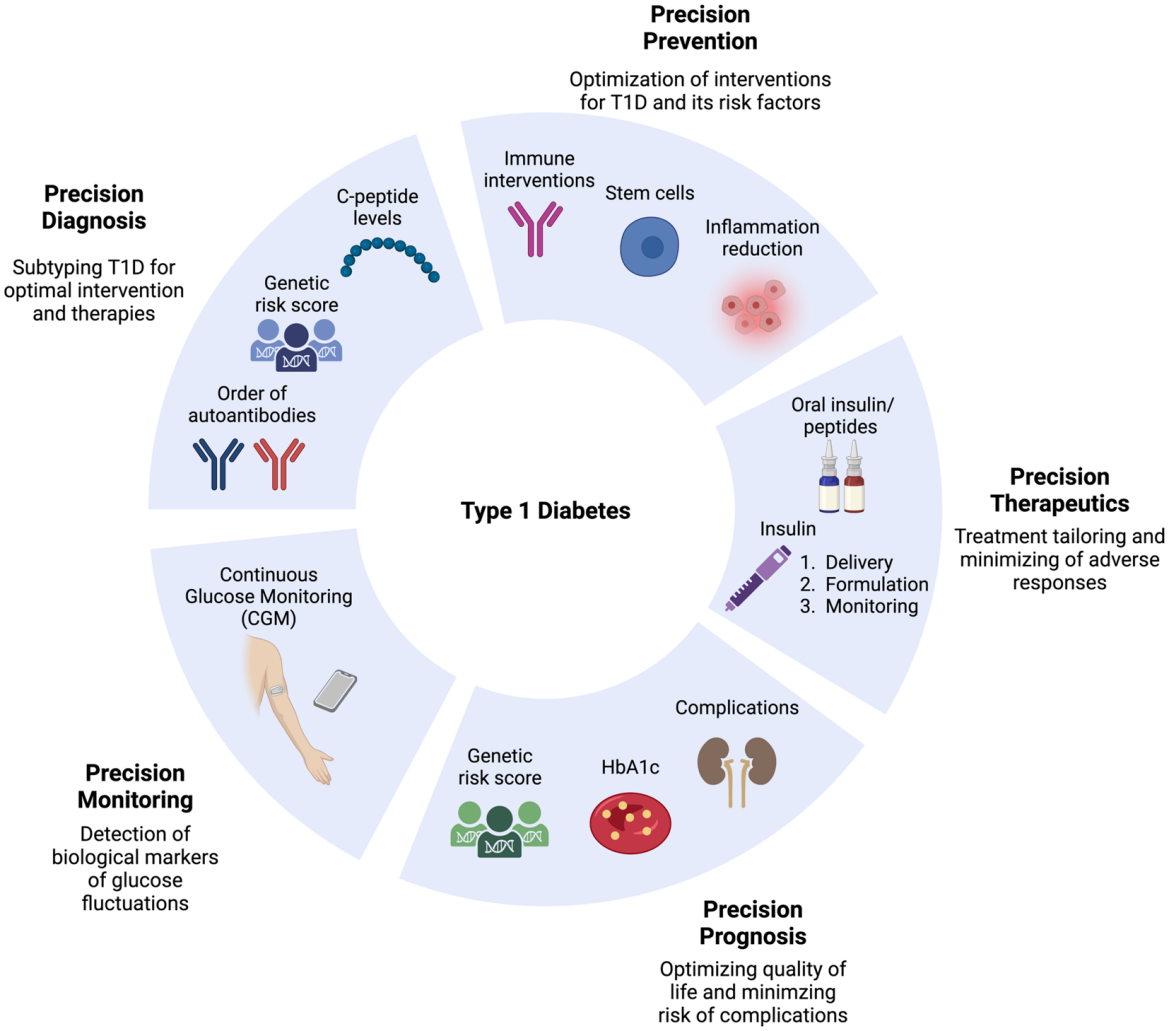
Five pillars of precision medicine in type 1 diabetes (T1D)–precision diagnosis, precision prevention, precision therapeutics, precision prognosis, and precision monitoring.

The approaches to precision medicine in type 1 diabetes are evolving with integration of technology, decision support systems, regulatory guidelines and patient engagement, all aspects that have been noted in the development of precision medicine in type 2 diabetes.^57^ Both type 1 diabetes and type 2 diabetes share aspects of genetic contribution to disease risk, although through quite different gene sets and fundamental etiology. The incomplete contribution of genetics to risk necessarily limits the utility of screening, as does the lack of diversity in genetic studies.^58^; in addition, there is limited information globally on the natural history of type 1 diabetes, autoantibody development and temporality, and progression to clinical disease in low prevalence and under-represented populations. However, knowledge of genetics provides significant insights into disease heterogeneity and potential for subtyping into more homogeneous groups of patients that can be used for improved treatment and prediction of disease progression. Ultimately, the implementation of “precision medicine” approaches, versus “classical medicine” approaches, involves using data analytics to enhance early prediction of islet autoimmunity, detection of those at high genetic and immunologic risk, and matching novel immune (or non-immune) interventions to delay or prevent disease; in those progressing to disease, precision monitoring would greatly reduce the occurrence of DKA at onset (Fig. 2).

### Screening

In type 1 diabetes, with genetic variation accounting for ~ one-half of the risk, screening that includes presence of autoantibodies has improved prediction of disease initiation and progression to clinical diabetes. Together, the concept of “staging” h

**Staging in type 1 diabetes**: is the concept of characterizing the disease in three components.

**Stage 1**: occurs when there is presence of two or more islet autoantibodies but normal glucose tolerance and no symptoms.

**Stage 2**: occurs with presence of two or more islet autoantibodies with abnormal glucose tolerance but no symptoms.

**Stage 3**: is the usual clinical diagnosis of type 1 diabetes.

as been proposed and embraced in type 1 diabetes,^59^ emerging from natural history studies of type 1 diabetes that extends the original concept that recognized the features of initiation, progression and clinical diagnosis.^15^ The concept of staging has been utilized as an approach for population screening,^60^ with specific recommendations that include use of genetic markers only in research and limited to HLA class II typing (that accounts for ~ 50% of genetic risk, or 25% of total risk), autoantibody screening in children < 10 years of age, and metabolic testing for estimation of the first phase insulin response. Screening in Germany has been highly informative for implementing an initial autoantibody screen,^61^ recruitment through an established health care system,^62^ and determining cost.^63^ In a parallel study in the USA, a pediatric screening program for celiac disease and type 1 diabetes has provided experience in a clinic setting, with both non-Hispanic white and Hispanic children involved,^64^ and experience gained in these and other studies.^65^ can provide guidance for approaches in more diverse and less-resourced populations. It should be noted, however, that screening costs currently vary across countries and, in particular, are dependent on specific health care delivery systems; thus, costs for screening may not be viable for most countries.^66^ although, in theory, screening could be expected to lower subsequent health care costs from reduced rates and severity of complications.

### Intervention

A major barrier in type 1 diabetes prevention is the availability of an appropriate intervention, where “appropriate” includes safety, affordability, quality of life, and absence of secondary effects; thus, the role of precision medicine in type 1 diabetes focused on identifying the target group of individuals who are likely to benefit from a specific intervention.^67^ A growing number of interventions at the islet autoimmunity stage (prior to clinical onset, or Stage 3) of type 1 diabetes have been developed and being used in clinical trials.^30^ It is likely that multiple intervention approaches will be required, as response to an intervention will depend upon the stage of beta cell destruction, the immune regulatory landscape, concurrent physiologic status and genetic variation, with the “endophenotype” grouping providing the appropriate interventions.

Progress in immune intervention has been led by the Fc receptor nonbinding anti-CD3 monoclonal antibody, teplizumab, in relatives of individuals with type 1 diabetes and high risk of disease.^68^ In a phase 2, randomized, placebo-controlled, double-blind trial (NCT01030861), a single 14-day course resulted in a delay (by ~ 24 months) in progression to clinical (Stage 3) disease, with annualized rates of type 1 diabetes occurrence reduced from ~ 36% in the placebo group to ~ 15% in the teplizumab group.^68^ In an extended 923-day median follow-up, the difference in time to diagnosis remained similar in the placebo group (~ 27 months) but was extended in the teplizumab group (~ 60 months), with 50% of the teplizumab group remaining disease-free.^69^ Of importance for future trials and applications of precision prevention are those characteristics that determine success in similar groups, as well as extensions to diverse at-risk populations in order to optimize improvements in metabolic responses and delay, or development, of type 1 diabetes with immune therapy.

### Prognostics and Monitoring

Key factors for prediction of outcomes for those with type 1 diabetes include traditional “biomarkers”,

**Biomarker**: is a measurable substance whose presence is associated with disease (type 1 diabetes), risk factor or environmental exposure. Biomarkers are typically measured in urine (non-invasive) or blood (invasive) to examine factors that may lead to risk or prediction of disease.

such as clinical characteristics of disease state, glycemic control, genetic propensity (whether based upon DNA or other ‘omic evidence or family history), social/cultural and physical environment, individual behavioral characteristics and access to care. Implicit in developing a precision medicine approach to prediction of outcomes (prognostics) is monitoring the state of disease at intervals. Improvements in monitoring, such as seen in continuous glucose monitoring, can have a major impact on prediction of development of type 1 diabetes.^70^ as well as prediction of subsequent risk of complications using metrics.^51,71^ As complications of type 1 diabetes represent the primary outcomes associated with treatment satisfaction, quality of life, morbidity and mortality, the availability of dynamic and immediate data on an individual’s physiologic state from wearables and other devices can be used to monitor health as well as guide active treatment.

## Global Implementation of Precision Medicine to Type 1 Diabetes

**Figure 3:**
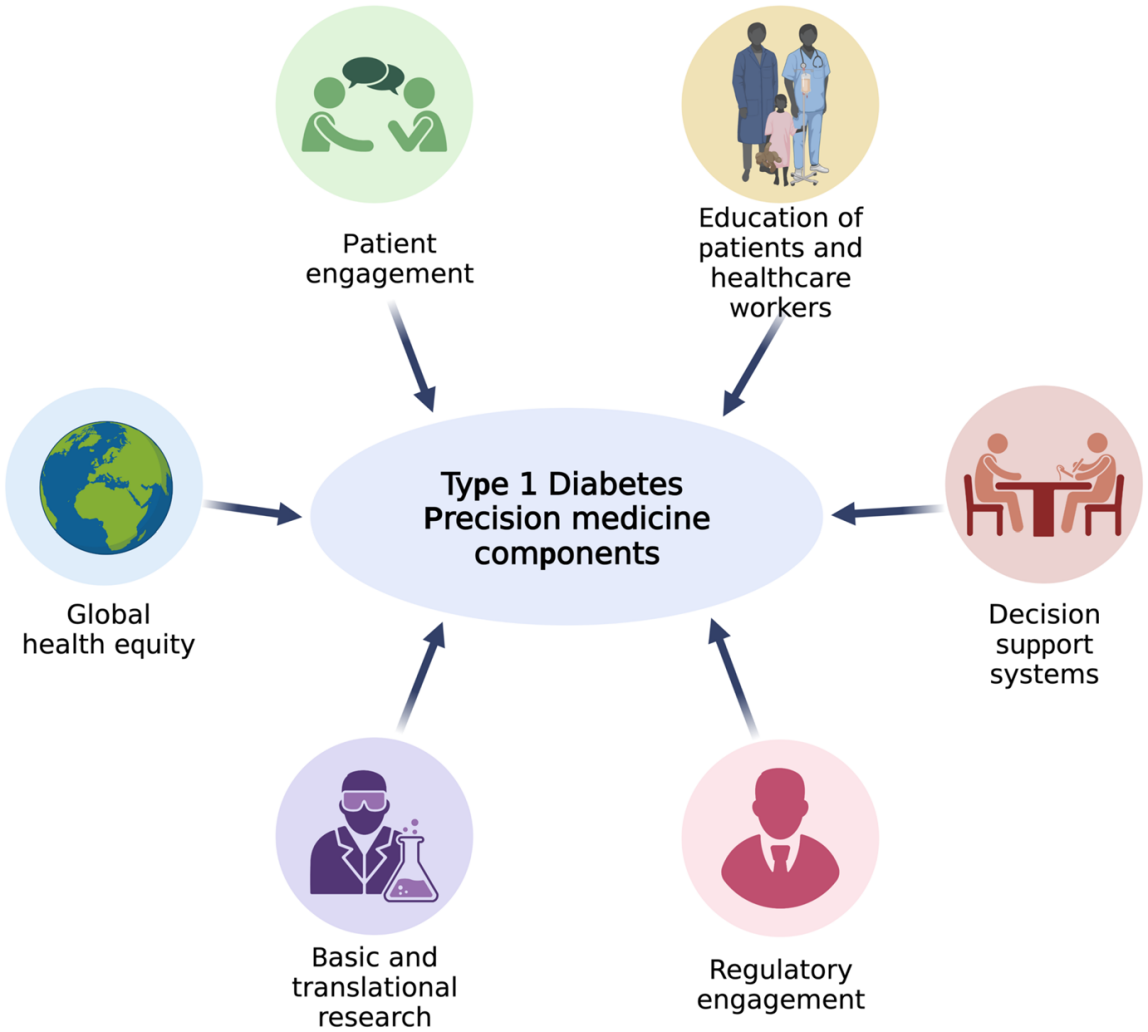
Components of precision medicine in type 1 diabetes that are beyond basic, clinical and population sciences, including patient engagement, education, decision support and global regulatory engagement.

While great advances are being made in understanding the genetic architecture of type 1 diabetes,^4,5^ development of immune-focused interventions,^68,69^ and miniaturization of wearables for monitoring,^71^ the translation of many advances in precision medicine applied to type 1 diabetes will require aspects not directly related to either basic science or clinical medicine (Fig. 3). Results from basic science, clinical and population sciences will need to be adopted by a heterogenous and fundamentally diverse process that will necessarily be tailored to each country (and, within countries, regional and local agencies).

Features of implementation of precision medicine in type 1 diabetes will necessarily involve patient engagement, educational systems that target healthcare delivery

**Health care delivery**: represents the visible function of a health system (e.g., a hospital or clinic) to both patients and the general public, involving patient flow and organization of services with respect to diagnosis and treatment of disease, and the promotion, maintenance and restoration of health.

(doctors, nurses, support staff) as well as patients, regulatory agencies (for those countries with reimbursement protocols), and mechanisms that will ensure health equity at a global level. Many of these issues have been noted previously.^20^; however, implementation will be dependent upon many societal and economic factors.

One of the “pillars” of precision medicine is precision diagnosis. In type 1 diabetes, it is thought that a portion of adults with type 1 diabetes may be misdiagnosed as having type 2 diabetes. A number of algorithms have been developed to aid in reducing misdiagnosis using standard clinical features with or without genomic information.^36,72,73^ In a retrospective study, a machine learning

**Machine learning**: is a part of artificial intelligence that builds a model based upon sample data in order to make predictions or decisions without explicit programming of a model. Machine learning algorithms perform tasks by having the computer imitate human behavior and learning.

algorithm was employed using ambulatory electronic medical records that included age, demographics, risk factors, symptoms, treatments, procedures, vital signs and available laboratory values.^74^ The machine learning algorithm identified age, BMI/weight, therapy history and HbA1c/blood glucose as the primary predictors of misdiagnosis. While these data are suggestive of machine learning approaches being useful in diagnosis, the available data and context is critical in assessing performance. Recent results from a study in Uganda,^75^ a less-resourced country, characterized lean versus non-lean individuals with “new-onset type 2 diabetes” with socio-demographic, clinical, biophysical and metabolic features with screening for islet autoantibodies. In this setting, 32% of subjects were lean (with diagnosis of type 2 diabetes), yet 6.4% of these had an autoimmune form (type 1 diabetes) based upon islet autoantibodies. Thus, translation of advanced (machine learning) approaches for precision diagnosis will be dependent upon the context of the population as well as the resources and data available.

In precision monitoring and management of type 1 diabetes, particularly in adolescents, blood glucose and insulin administration can be challenging. Data science-driven approaches, such as use of machine learning algorithms, have been tested to identify risk of suboptimal self-management.^76^ Youth from the Vanderbilt Eskind Pediatrics Diabetes Clinic (13–19 years of age) participated if they had a smartphone and could use a Bluetooth blood glucose meter with implementation of an ecological momentary assessment (EMA) model coupled with a machine learning algorithm. The EMA model integrated with the machine learning algorithm was able to predict those with deviations from appropriate management; however, the major factor in achieving improved self-management behavior of adolescents with type 1 diabetes was related to social determinants of health

**Social determinants of health**: are the conditions in which people are born, grow, live, work, and age that affect and shape health. There are numerous factors that constitute social determinants of health, including socioeconomic status, education, neighborhood and physical environment, social support networks, and access to health care..

The psychosocial component and the methods used to assess appropriate management (wearable technology) may represent another major barrier globally for implementation of precision medicine in type 1 diabetes.

Implementation of precision medicine in type 1 diabetes will involve education at many levels, focused on how the clinician interacts with the patient and the return and action on the available data from a variety of sources. In many western societies, extensive electronic health record systems exist, often with elaborate biobanks of samples that permit extensive use of genomics to characterize disease risk and response to therapeutic agents. The IGNITE network evaluated several clinical decision support systems to aid clinicians and patients interpret and act on genomic data.^77^ All projects included feedback from stakeholders, identified “local champions”, and included training for clinicians and stakeholders. The IGNITE experience identified several key “lessons” for integrating genomic data into a clinical service including (1) a variety of strategies will be necessary to tailor genomic medicine to a specific practice and environment, and (2) providing patient genotypes in an electronic health record format to guide therapeutic guidance is complicated to implement as it relies on disease-specific and patient-specific needs and preferences. While this process was conducted in the USA, other countries will have different health care systems, tracking and practices that would make the strategies tested either unlikely or cost-prohibitive.

## Cost Effectiveness of Precision Medicine

The cost of implementing precision (or personalized) medicine and its impact on quality of life is an emerging area of research. This research is challenging due to global differences in burden of disease (whether primary impact is on infectious versus chronic disease), existence and availability of diagnostic tests and treatments, and education/training of health care providers as well as patients. Recent work has focused on systematic reviews of the literature, often in the area of oncology, yet there are consistent findings related to the impact on cost and potential cost savings of precision medicine, as well as barriers, that have emerged.

Much of the focus of precision medicine has been on the use of genomics technology for diagnosis, treatment and evaluation of treatment response, especially in the field of oncology. Although other technologies are being applied to augment genomic profiling in precision medicine, including gene therapy for specific disorders, the concept remains genomic. The assessment of evidence of economic value of precision medicine approaches often has utilized the incremental impact on quality-adjusted life years (QALYs). Using an available cost-effectiveness analysis registry, an analysis of studies found that a majority (72%) of precision medicine tests did provide improved health, but at a higher cost,^78^ with only 20% of the tests predicted to save money. In this series, primarily based on data from the USA, the tests used genetic or molecular information that is appropriate for testing of germline or somatic mutations, and excluded conditions that have relatively few genetic causes. Although results were based in a high-income country, with extensive data collection systems, the analysis found that only 25% of available tests and 20% of tests with clinical utility had associated cost-utility data,^78^ illustrating a major gap in knowledge across both high- and low-income countries.

A more recent global evaluation of cost-effectiveness of precision medicine still had the majority of data from countries in Europe (31%) and North America (28%) and focused on cancer (43%) and cardiovascular disease (28%), outcomes of westernized societies.^79^ Over 70% of the studies analyzed concluded that the cost-effectiveness of precision medicine was equivalent to usual care, although the “willingness to pay” thresholds varied significantly. Thus, the implementation of precision medicine in a country is dependent upon the money per QALY, which is dependent upon the fiscal health and priority of the country. The key factors influencing cost-effectiveness were prevalence of the genetic “condition” in the population, the costs and accuracy of genetic testing and treatment, and the likelihood of complication or mortality from the condition. Using a different metric, the “net monetary benefit” (NMB) of precision medicine, similar outcomes were identified.^80^ in the large heterogeneity across conditions in upper-middle- or high-income countries, with the greatest benefit in cancer, yet still at a high cost to improve health. As was illustrated in this and other analyses, there is difficulty in assessing cost-effectiveness thresholds in each country, in part due to limited data, difference in perspectives of what the threshold represents (society’s willingness to pay for increases in health versus the cost of health care spending), and how the threshold should be calculated. Again, the results suggest that the health benefits of precision medicine are more costly than, but similar (or slightly lower) than the health benefits of other interventions.

The majority of cost-effectiveness evaluations of precision medicine have been conducted through systematic reviews of published literature that are limited in terms of low-income (as well as middle-income) countries. Although this is due, in part, to the limited economic evaluations of precision medicine available in low-income countries, a recent report on precision medicine in oncology focused on Nigeria and Nepal, providing important insights applicable to other low-income countries and conditions.^81^ In Nigeria, with Africa’s largest economy and population, ~ 3% of the Gross Domestic Product was spent on healthcare, similar to the ~ 4.5% in Nepal, more than the ~ 2% of India, but much less than ~ 20% of the USA. Specific barriers to implementation of precision oncology in Nigeria and Nepal were identified as both structural (lack of funding health systems for availability, affordability and acceptability of health services to the population) and technological (high cost of equipment and genomic diagnostic devices). In oncology, genomic information on “driver mutations” are known primarily in those of European ancestry, making application to low-income, non-European ancestry populations limit transferability of testing and reduced “precision” of molecular diagnoses. Compounding the structural and technological barriers of implementation of precision medicine is the avoidance of use of precision diagnostic tests because of lack of training in interpretation of test results. Although this factor is not limited to low-income countries, it is more prevalent due to lack of educational infrastructure and experience with new technologies. Together, these factors lead to health care system limitations, physician resistance, and patient unawareness.

The cost-effectiveness of precision medicine in low-income countries has limited data, but significant barriers (particularly in oncology) have been identified. It should be noted that the progress made in precision medicine in oncology has been considered the “role model” for precision medicine in other conditions. The status of diabetes treatment in low- and middle-income countries has been reviewed recently, as ~ 80% of adults with diabetes (typically type 2 diabetes) live in these countries.^82^ Fewer than 10% of those with diabetes received comprehensive, guideline-recommended treatment, as the proportion of those eligible actually receiving treatment varied with income and region – coverage of glucose-lowering medication was ~ 40% in low-income countries, ~ 45% in lower-middle-income countries, and ~ 64% in upper-middle-income countries. These results highlight the need to improve treatment delivery for glucose lowering and reduction in complications risk among those living with diabetes in low- and middle-income countries. These goals form the basis of diabetes medicine without advanced technology, serving as a first-line approach to improved clinical outcome through “usual care”, prior to the application of precision medicine approaches.

## Summary

Precision medicine as applied to type 1 diabetes is evolving rapidly along all of the “pillars” of precision medicine. There are advances being made in diagnosing type 1 diabetes accurately through use of standards of care, improved testing and inclusion of novel biomarkers. At the same time, it is being recognized that type 1 diabetes is, itself, heterogeneous and stratification into subclasses of disease, particularly across global populations, may enable improved targeted treatments to improve glycemic control and reduce risk of complications, with improved long-term quality of life. Improvements in monitoring the physiologic state are being applied to all aspects of precision medicine (diagnostics, therapeutics, prevention and prognostics) and serves as a cross-cutting application of technology and data science to improve health. These improvements in monitoring are needed globally, to benefit all populations, and the process to reduce cost and simultaneously provide an optimal decision support system represents a major barrier to implementation. Finally, while insulin is the sole treatment for type 1 diabetes, there have been major advances in developing interventions to delay onset of type 1 diabetes. It is anticipated that multiple interventions will be required, tailored to specific subgroups of at-risk individuals, with the subgroups defined by precision medicine principles. In the future, precision medicine applied to type 1 diabetes would enable early identification of those at risk, classification into subgroups for intervention, and optimization of treatment in those who develop disease to reduce risk of complications. Effectively, one needs to address the needs of the individual and their features of risk (susceptibilities) and target medical intervention before, and not after, disease onset. This effort will necessarily require an integration of teams to develop a plan for the implementation of precision medicine for type 1 diabetes. To ensure this effort is beneficial to all individuals at risk, additional research is needed to improve ancestral and geographic representation into our precision prediction models.

## Data Availability

There are no primary data included in this manuscript; it is a “perspective” and summarizes the current status of precision medicine in type 1 diabetes.
